# Syncope as a health risk for soldiers - influence of medical history and clinical findings on the sensitivity of head-up tilt table testing

**DOI:** 10.1186/s40779-015-0062-1

**Published:** 2015-12-02

**Authors:** Hans-Joachim Gilfrich, Lena Marie Heidelmann, Franziska Grube, Hagen Frickmann, Sven Andreas Jungblut

**Affiliations:** The Practice of Dr. Jungblut, Frankfurt/Main, Germany; The Flight Medicine Clinic at Fassberg, German Armed Forces, Faßberg, Germany; The Department of Tropical Medicine at the Bernhard Nocht Institute, German Armed Forces Hospital of Hamburg, Hamburg, Germany; The Department of Microbiology, Virology and Hygiene, University Medicine Rostock, Rostock, Germany

**Keywords:** Syncope, Head-up tilt testing, Predictor, Soldiers, Assessment, Hypotension

## Abstract

**Background:**

Syncope is a relevant health problem in military environments. Reliable diagnosis is challenging. Tilt table testing is an important tool for syncope diagnosis. The aim of this study was to determine whether signs such as prodromal symptoms, co-morbidity, frequency of syncopal events, body length, body mass index, and electrocardiography abnormalities can be used to predict the success of tilt table testing at diagnosing syncope.

**Methods:**

Data from 100 patients with histories of syncope or pre-syncope, who were diagnosed using head-up tilt table testing, were retrospectively analyzed in a cross-sectional analysis. The diagnostic procedure was based upon a modified version of the Westminster protocol without any pharmacological provocation.

**Results:**

Patients showing pathological reaction patterns during tilt table testing suffered from prodromal symptoms, such as dizziness and sweating, significantly more often. The patients reported more injuries resulting from syncopal events and more previous syncopal events, and the prevalence of co-morbidity was greater among patients presenting negative findings during tilt testing. An asthenic-leptosomal physique was not confirmed as a risk factor for syncopal events as is the case for idiopathic arterial hypotension. However, patients with pathological reaction patterns during tilt table testing were significantly taller. This finding was detected for both females and males. No significant predictors were found in the electrocardiogram (ECG) patterns of patients showing syncope during tilt table testing.

**Conclusions:**

Frequency of prior syncope and prodromal symptoms, and increased body length with an otherwise good state of health influence the predictive value of tilt table testing for syncope diagnosis. In particular, if these factors are present, tilt table testing should be considered part of the diagnostic algorithm for soldiers with recurrent syncope.

## Background

Syncope has been defined as a transient, self-limited loss of consciousness based on a transient global cerebral hypoperfusion that usually leads to falling with rapid onset and spontaneous subsequent complete and prompt recovery [1]. As with civilians, military service members can be affected. Syncope commonly affects personnel that stand in ranks during military ceremonies, leading to disruption of the ceremonies and embarrassment for the individuals involved [2]. The identification of a possible sudden incapacitation during military flight is essential to maintain high aviation safety. Sudden incapacitation can be associated with syncope [3]. Medical assessment, including an evaluation of incapacitation risk, is mandatory for flight personnel who wish to obtain the medical certificate that is required for maintaining pilot licenses in European aviation [3, 4].

**Table 1:** Patient clinical data

Item	Data
Total number of patients	100
Male [*n* (%)]	45 (45 %)
Female [*n* (%)]	55 (55 %)
Average age (year)	53
Minimum/maximum age (year)	15/88
Syncopal events	
Minimum	0
Maximum	40
Mean value	4.43
Standard deviation	5.98
Patients without medical history of syncope	12
Accompanying diseases	
Arterial hypertonus	19
Diabetes mellitus	9
Atherosclerosis of the carotic blood vessels and/or central nervous system blood vessels	9
Coronary heart disease	8
Disturbed heart rhythm	7
Diseases of the thyroid gland	6
Heart insufficiency New York Heart Association I-II	4

The calculated incidence rate of syncope within the armed forces of the United States of America is 7.2 cases per 1,000 person years. Four percent of syncopal events are associated with physical injury. Frequency of syncope in association with vaccination ranges from 4.4 to 14.1 events per 100,000 immunization episodes [5].

Head-up tilt testing is the gold standard for the diagnosis of neurocardiogenic syncope (also called vasovagal syncope). The procedure allows five types of reaction patterns to be distinguished: neurocardiogenic syncope (including vaso-depressive, cardio-inhibitory and mixed subtypes), dysautonomic syncope, postural tachycardia syndrome (POTS), cerebral syncope, and psychogenic syncope [6].

However, head-up tilt testing remains a technically demanding and time-consuming diagnostic procedure despite its advantages [7, 8]. Thus, it is not routinely performed to diagnose syncopal events, although the pathophysiological background of approximately half of etiologically unclear syncopal events can be determined using head-up tilt testing [9]. Accordingly, the search for predictors of the efficiency of using this method for the differential diagnosis of syncope appears useful.

This retrospective assessment was performed to identify features in the clinical history or diagnostic findings that can help to identify patients with syncope who are likely to obtain diagnostic benefit from head-up tilt testing. Parameters including prodromal symptoms, accompanying diseases, frequency of syncope, height, body mass index, and electrocardiogram(ECG)-findings were analyzed to assess their prognostic value for the onset of syncopal events during head-up tilt testing.

Although some parameters (e.g., height) are known factors for tolerance time to tilt, literature results are partly inconsistent, as detailed in the discussion section. This proof-of-principle analysis was designed to observe whether such factors are reproducible and to strengthen described associations based on additional data. In this way, the aim of this study was to identify major and minor criteria that might suggest diagnostic benefit from head-up tilt testing that might be worth assessing in sufficiently powered future studies.

## Methods

### Patients

Data obtained from 100 consecutive patients (45 male, 55 female) were anonymously and retrospectively assessed in this cross-sectional assessment. The mean age of the population (± SD) was 53 (±19 years), and the median age was 56. The diagnostic setting was previously described in detail [10] and is summarized below (Table 1).

The youngest patient was 15 years, and the oldest was 88 years. All patients were referred to the hospital due to a history of suspected neurocardiogenic syncope. History of syncope or pre-syncope was the main criterion for inclusion. Exclusion criteria included the following illnesses: hypertrophic obstructive cardiomyopathy, higher-grade stenosis of the aorta, pulmonary hypertonus, bradycardia (sick sinus syndrome, second- or third-grade atrioventricular block), tachycardia (supra-ventricular tachycardia, ventricular tachycardia, Torsade-de-pointes-tachycardia), haemodynamically relevant stenoses of the central blood vessels, heart insufficiency of the New York Heart Association stages III-IV, and neurological disorders that can cause disturbed orthostatic regulation (Shy-Drager’s syndrome, Parkinson’s disease, Encephalitis disseminata, Guillain-Barré’s syndrome, and narcolepsy).

The frequency of syncopal events ranged between a minimum of zero and a maximum of 40 syncope events. The mean value was approximately 4.43 ± 5.94 (SD) events. Twelve patients were free of proven syncopal events. These patients showed pre-syncope or typical symptoms of disturbed orthostatic regulation.

The following accompanying diseases were present: 19 patients had arterial hypertonus, 9 patients had type II diabetes mellitus, 9 patients had atherosclerosis of the carotic blood vessels and/or blood vessels of the central nervous system, 8 patients had coronary heart disease, 7 patients had known disturbances of the heart rhythm, 6 patients had diseases of the thyroid gland and 4 patients had a heart insufficiency of a lesser grade than New York Heart Association III. Approximately 54 patients did not present any accompanying disease (Table 1).

**Fig. 1:**
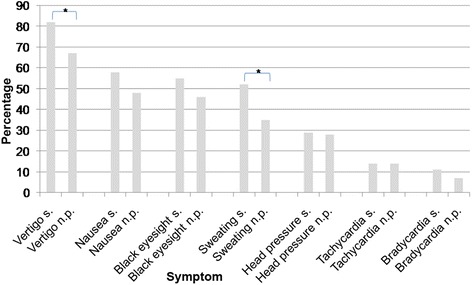
Prodromal symptoms. s. Syncope during head-up tilt testing; n.p. No pathological findings during head-up tilt testing. *P < 0.05

All patients were asked about typical prodromal symptoms before the onset of syncopal events. In particular, the incidence of vertigo, nausea, black eyesight, sweating, headache or head pressure, tachycardia, bradycardia, and injuries was determined. The height and body mass index of all study participants were measured. An electrocardiogram with 12 channels was obtained using a MAC 1200 ST ECG device (GE Healthcare, Chalfont St Giles, UK), and a routine blood analysis was performed. Blood was drawn in the morning prior to head-up tilt table testing.

### Test conditions

Using the head-up tilt device (which was electronically controlled via a hydraulic system), the intended tilt angle could be adjusted and returned to the starting position within 15–20 seconds. A remote control device allowed the intended tilt angle to be programmed directly. The tilt could be controlled using an external protractor that was attached to the table. The head-up tilt device was self-designed as described [10]. Electrocardiographic (ECG) analysis was continuously performed throughout the analysis. The ECG analysis was recorded using chart paper. Blood pressure was measured in parallel according to Riva-Rocci [11]. Blood pressure cuffs (Erkarapid classic Germany; Erka. Kallmeyer Medizintechnik GmbH & Co. KG, Bad Tölz, Germany) and Littmann classic II S.E. stethoscopes (3 M Deutschland GmbH, Neuss, Germany) were used. The test was performed in a warm, quiet room under dimmed light to avoid unintended stimulation of the nervous system. The testing took place between 9 and 12 AM. The patients were asked to abstain from food and liquids for at least 6 hours prior to the test to avoid postprandial orthostatic hypotonia. Nicotine consumption was also prohibited for 6 hours prior to the testing. The administration of cardiovascular-acting drugs was ceased for at least 5 half lives of the respective drug before the testing. All patients were informed about the procedure; a standardized questionnaire and a detailed talk were presented to prepare for the testing.

The applied head-up tilt testing protocol was based upon a modified version [12] of the “Westminster protocol” [7], the first recommended protocol for medical head-up tilt testing. After fixing the patient at the table and applying electrocardiography electrodes and blood measurement equipment, the patients were allowed to rest for twenty minutes in a horizontal position. An electrocardiography sheet was printed at the end of the rest period. The patient was moved within 15 seconds during the following tilt period into a 70° angle in relation to the initial position, with the head up. An additional electrocardiography sheet was printed one minute after the new position was reached. This test was ended after 45 minutes if no symptoms occurred. Finally, the patient was then returned to the horizontal position and allowed to rest for five minutes. A further electrocardiography sheet was printed at the end of the analysis. In cases of syncope or pre-syncope, the patients were returned immediately to the horizontal position. In such cases, electrocardiography sheets were printed continuously until the cardiovascular parameters returned to normal values.

Patients who suffered from neurocardiogenic or orthostatic syncope during the head-up tilt testing were compared with patients who remained asymptomatic during the procedure. Patients with postural tachycardia syndrome during head-up tilt testing were not included in either of the two groups to guarantee clear discrimination.

### Descriptive statistics

All results were described using representative parameters, such as median, mean, and standard deviation. The quantitative results of the groups were compared using Student’s *t*-test. The measured values were subjected to normality testing as a prerequisite for t-testing. Non-parametric (Mann–Whitney) testing was performed in case normality could not be confirmed. Values of *P* < 0.05 were considered significant. For data obtained using contingency tables, Fischer’s exact test with Yate’s continuity correction was applied to calculate two-sided *P* values. Values of *P* < 0.05 were considered significant.

### Ethical considerations

Ethical clearance was not required for this assessment in accordance with German law. Anonymous patient data were retrospectively assessed. No medical procedures were performed for study purposes. Accordingly, the project did not fulfill the criteria for description as a scientific project involving humans according to § 9.2 of the Law of the Association of Hamburg Physicians (“Hamburgisches Kammergesetz für Heilberufe”), and ethical counseling was not required according to § 15.1 of the Professional Guidelines of Hamburg Physicians (“Berufsordnung für Hamburger Ärzte und Ärztinnen”). Due to the anonymous nature of the assessment, German data protection law (“Bundesdatenschutzgesetz”) was not applicable.

## Results

### Results of head-up tilt testing

Head-up tilt testing of 100 patients was assessed; of these, 56 patients showed a non-pathological result. Fourteen patients suffered from orthostatic dysautonomia with syncope during the test. Neurocardiogenic syncope was detected in 20 patients. An additional 10 patients presented postural tachycardia syndrome (POTS).

In summary, syncopal events were induced in 34 % of the patients, and a pathological reaction pattern was induced in 44 % of the patients by head-up tilt testing. In comparison, non-pathological findings were present in 56 % of the patients. The 10 % of patients who suffered from postural tachycardia syndrome will not be discussed further in this paper.

### Prodromal symptoms

The medical histories of typical prodromal symptoms of syncopal events revealed the following results for the group without pathological findings during head-up tilt testing: 67 % suffered from vertigo, 48 % had nausea, 46 % had black eyesight, 35 % presented sweating, 28 % experienced headache or head pressure, 14 % exhibited tachycardia and 7 % exhibited bradycardia. Thirty percent of these patients had at least one injury (a significant contusion, bone fracture, cut, commotio cerebri, or contusio cerebri) as the result of falling to the ground during a syncopal event. Head injuries represented approximately 47 % of all injuries.

Patients with a syncopal event during head-up tilt testing reported vertigo in 82 % of the cases as a typical prodromal symptom. Nausea was reported by 58 %, black eyesight by 55 %, sweating by 52 %, headache or head pressure by 29 %, tachycardia by 14 % and bradycardia by 11 %. Approximately 47 % of these patients presented injuries that were caused by accidents during syncope. Head injuries represented 55 % of all injuries.

**Fig. 2:**
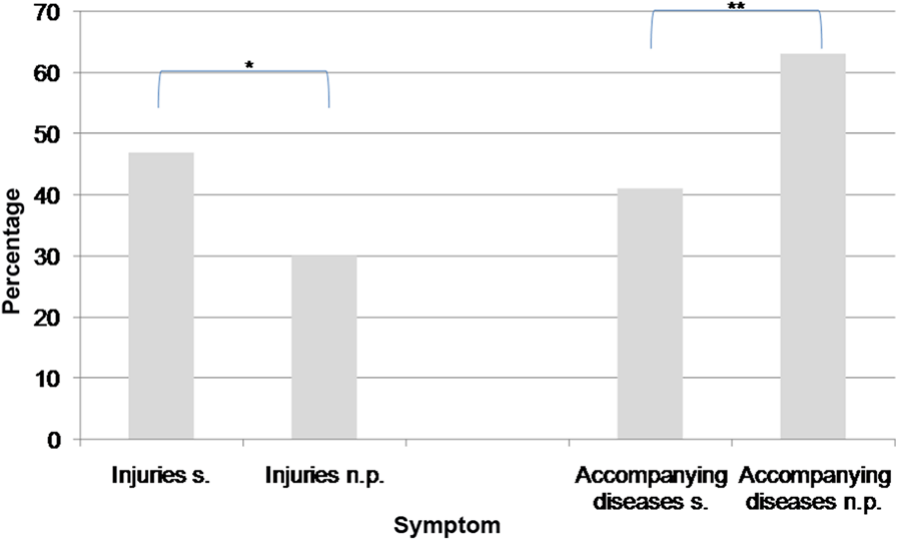
Injuries during syncopal events and accompanying diseases. s. Syncope during head-up tilt testing; n.p. No pathological findings during head-up tilt testing. *P < 0.05, **P < 0.01

Significance was found for more frequent occurrence of the prodromal symptoms sweating and vertigo and of injuries in patients who experienced a syncopal event during head-up tilt testing (Figs. 1 and 2).

### Accompanying diseases

The distribution pattern of accompanying diseases showed significantly more frequent occurrence of accompanying diseases in the group without pathologies during head-up tilt testing than in the group with induced syncope (*P* < 0.003). Whereas 63 % of the asymptomatic patients had at least one accompanying disease, only 41 % of the patients in the group with positive test results had at least one accompanying disease (Fig. 2).

### Total count of syncopal events

**Fig. 3:**
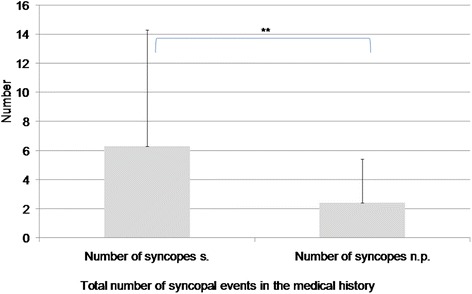
Total count of syncopal events. s. Syncope during head-up tilt testing; n.p. No pathological findings during head-up tilt testing. **P < 0.01

The average patient not experiencing syncope during head-up tilt testing had previously suffered from 2.4 ± 3 (SD) syncopal events during his or her life. However, patients experiencing syncope during testing reported histories of 6.3 ± 8 (SD) syncopal events in their medical history. This highly significant difference (*P* < 0.001) remained stable even after 3 patients with extremely high numbers of previous syncopal events were removed from the analysis. After this correction, the mean value for patients with syncope during head-up tilt testing was 4.059 ± 3 (SD) (*P* < 0.002) (Fig. 3). No significant difference in the total number of syncopal events was found between male and female patients.

### Height and body mass index

The average height of female patients not experiencing syncope during head-up tilt testing was 162 ± 6 (SD) cm, whereas that of asymptomatic males was 175 ± 6 (SD) cm. The mean value of height in symptomatic females was 167 ± 5 (SD) cm, whereas that of symptomatic males was 180 ± 7 (SD) cm. Patients who developed syncope during testing were significantly taller (*P* < 0.002 for females, *P* < 0.032 for males).

In patients without syncope during head-up tilt testing, mean body mass index was 24 ± 4 (SD) kg/m^2^ for females and 27 ± 3 (SD) kg/m^2^ for males. In patients with syncope, mean body mass index was 24 ± 3 (SD) kg/m^2^ for females and 26 ± 3 (SD) kg/m^2^ for males. No significant results were found when comparing patients who were symptomatic or asymptomatic during tilt table testing.

### Electrocardiography

Intermittent cardiac arrhythmia was documented for three patients in the group without syncope during testing. The other patients in this group showed a normal sinusoidal rhythm. Without exceptions, the patients in the syncope group showed a sinusoidal rhythm in the 12-channel-electrocardiogram. During the ECG analyses, special attention was given to PQ-time as a possible predictor of bradycardia-like disturbances of heart rhythm and increased vagal tone. However, no significant differences were found between asymptomatic and symptomatic patients. The mean value of PQ-time was 166 ± 51 (SD) ms in patients without detectable syncope and 167 ± 33 (SD) ms (*P* = 0.880) in patients with syncope during head-up tilt testing.

## Discussion

During military deployment, syncope is the most frequent reason for the referral of US-American soldiers to cardiology after ischemic evaluation and arrhythmia/palpitations [13]. Injuries associated with syncopal events are infrequently registered in soldiers [5].

In aviators, syncope is particularly problematic because syncopal events during flight can impair safety, possibly causing loss of the airplane and the crew’s life [3, 14]. In a previous study, vasovagal syncope and heart rhythm-associated syncope were found to be correlated with the sudden incapacitation of aviators [3]. Multi-pilot requirements can ensure higher aviation safety [3]. In a systematic review, the incidence of sudden aviator incapacitation was found to lie in the range of 0.19 to 0.45 times/10^6^ flight hours [15]. Another study reported 39 in-flight incapacitations for U.S. airlines between 1993 and 1998. These incapacitations included 9 cases of loss of consciousness, 4 of which were due to vasovagal syncope and one of which was due to neurogenic syncope [16]. Therefore, screening military aviators for syncope is important to prevent in-flight incapacitation.

This study was designed to identify factors that predict positive tilt table testing results in patients with syncope. The prognostic significance of the examined parameters regarding the sensitivity of tilt table testing as a diagnostic tool for patients with syncope was very heterogeneous. The applied modified Westminster protocol is considered especially useful for syncope diagnosis without the underestimation of cardioinhibitory responses [12].

The results presented here extend the current knowledge on the subject. Nausea, vertigo, sweating, headache or head pressure, epigastric symptoms and disturbed eyesight are typical prodromal symptoms of syncope [17]. History of syncope without heart disease accompanied by dizziness, nausea and diaphoresis is considered predictive of neurocardiogenic syncopal reactions [18]. Patients with a history of syncope often report visual blurring, dysesthesia, sighing dyspnea, tremor in fingers and diaphoresis. Sensations of hot flashes immediately before syncope are positively correlated with positive head-up tilt testing results. In contrast, the predictive value of clinical questionnaires appears negligible [19]. Hypertension is negatively correlated with head-up tilt testing results [12]. Low blood pressure and increased plasma renin levels predict orthostatic intolerance in dehydrated subjects [20]. The lack of an early sympathetic over-reactivity that leads to an increase in heart rate also predicts negative head-up tilt table test results [21]. The prediction of a forthcoming syncope during head-up tilt testing based on constant measurement of blood pressure and heart rate is possible with acceptable sensitivity and specificity [22]. The measurement of baroreceptor sensitivity and heart rate variability using power spectral analysis might contribute to an increased sensitivity of tilt testing in children [23]. The rate of positive results found during passive tilt testing can be tremendously increased by chemical agents, such as sublingual nitroglycerine. This agent appears to effectively unmask syncope with only a minor decrease in specificity [24]. However, reactions to nitroglycerin are partially age-dependent, and a more gradual decline of blood pressure is found in elderly patients [25]. Gender, presence of structural heart disease, baseline heart rhythm and presenting symptoms before the test were found to be of no important predictive value [26].

The medical history of prodromal symptoms yields very important hints concerning the causes of syncope. Nevertheless, approximately 30 % of syncopal events occur without any prodromal symptoms [27]. If typical prodromal symptoms are absent, obtaining the correct diagnosis is much more difficult [28].

In the analysis presented here, significant differences were found for vertigo and sweating between patients who expressed syncope during head-up tilt testing and patients who did not.

Further, injuries due to syncopal events were significantly more frequent in patients with positive test results. This finding agrees well with previous reports [29]. The high percentage of serious injuries found here for both groups underlines the importance of accurate diagnoses and therapeutic strategies for syncope patients. Other authors reported frequencies of injuries per syncopal event of up to 35 % in the elderly. Serious injuries are defined as bone fractures, subdural hematomas and traffic accidents caused by syncope. The proportion of such serious events is 17 % [30, 31].

Importantly, no significant differences were identified for nausea, headache or head pressure, and black eyesight. Tachycardia and bradycardia were rarely experienced by patients with negative or positive head-up tilt testing results as prodromal symptoms. No significant difference was found between the two groups in this respect. However, this does not necessarily mean that these symptoms are not present. Due to adaptation, the patients might simply be unaware of these symptoms. This lack of awareness renders tachycardia and bradycardia of little use as diagnostic criteria based on medical history.

Comparison of the accompanying diseases also revealed significant differences between the patients yielding positive and negative test results. An increasing number of accompanying diseases appears to be negatively correlated with the number of negative head-up tilt testing results. Increasing age is usually associated with an increasing number of chronic diseases. Indeed, previous analyses have shown that the risk of syncopal events that are not reproducible by head-up tilt testing is especially increased in elderly patients. Possible reasons include age-related physiological changes and an increasing number of chronic diseases and the associated pharmaceutical therapy [32]. In contrast, young age is considered an independent predictor of positive results during head-up tilt testing [29]. Other authors deny the predictive value of age. However, it is widely accepted that syncope typically occurs in young patients during earlier stages of testing than in older patients [26]. Nevertheless, head-up tilt testing shows satisfying results for the differential diagnosis of etiologically unclear syncope in patients aged over 65 years, and the results are even more satisfying in patients aged over 80 years [32].

Next to its effects on the reliability of tilt testing, age also influences the occurrence of syncope. As demonstrated in a recent review [33], the average incidence of syncope increases markedly in patients older than 70 years. Of note, studies addressing orthostatic intolerance in non-clinical subjects, both generally and for soldiers in particular [2, 5, 13, 14], usually analyze much younger cohorts than that assessed here. Accordingly, this analysis might overestimate the overall sensitivity of head-up tilt testing, and this sensitivity might be considerably lower in younger patients.

The finding that patients with a high number of syncopal events in their medical history are more likely to show syncope during tilt testing is consistent with previous literature [9]. A history of neurocardiogenic syncope is positively correlated with the results of tilt testing [12, 26]. In addition, the risk of remitting syncope is significantly increased if the number of previous syncopal events is raised from one to three. This risk is further influenced by the frequency of the events. Patients who have experienced repeated syncope over a short period present a higher risk than patients with longer periods between syncopal events [34–36]. The probability of experiencing further syncopal events after a single event is approximately 30 % [37].

The identification of physiognomic differences between patients with positive and negative head-up tilt testing results was a further aim of the study. The two groups were different in height. Patients with syncope induced by head-up tilt testing were significantly taller than patients with negative test results. This was true for both male and female patients. In contrast, no significant difference was found in body mass index for both men and women. Although no significant difference was found between the groups, patients with syncope were likely to have body mass indices at borderline obesity levels in this study, irrespective of the head-up tilt testing results. An asthenic-leptosomal physique (which is common in patients with arterial hypotonia) was not indicated as a possible predisposition based on the data obtained here. In contrast, other authors have reported a negative correlation between body mass index and head-up tilt testing responses [12].

PQ-time as a possible indicator of bradyarrhythmic heart rhythm disturbances and increased vagal tone was of special interest while interpreting the 12-channel-electrocardiogram charts. In particular, the cardioinhibitory variant of neurocardiogenic syncope has been reported to be associated with sinusoidal bradycardia up-to asystolia of more than 30 seconds or II° atrioventricular blocks [38]. Further, the presence of a junction rhythm is an independent predictor of positive head-up tilt testing results [29]. However, we did not observe any prolongation of PQ-time, and the symptomatic and asymptomatic patients during head-up tilt testing did not show any significant difference. Admittedly, the meaningfulness of the results is limited. Permanent electrocardiography measurement would have been a more meaningful method, an undeniable limitation of the study.

Of note, short time between the last syncope and head-up tilt testing is considered a further predictor of positive test results [29]. However, this element of the patients’ medical histories was not analyzed in this study.

Restriction of food and water intake might have affected the probability of positive head-up tilt testing results due to dehydration. These restrictions were applied to standardize the test conditions.

Head-up tilt testing was performed in the morning to ensure the standardization of this proof-of-principle analysis. The low number of participants did not allow the patients to be divided into groups for analyses at different times of day. Several studies suggest that the vasovagal response to head-up tilt exhibits a circadian rhythm, showing a higher frequency of syncopal episodes in the morning [39–43]. Therefore, head-up tilt testing was performed during the morning to increase the likelihood of obtaining positive test results.

In future studies more differentiated analyses should be performed, generally dividing the volunteers into male and female groups. The low number of volunteers used in this proof-of-principle study does not allow meaningful assessments regarding this issue. Therefore, gender stratification was only performed for body mass index (BMI) and body height, which are considerably different in men and women and could be calculated for all volunteers. As previously observed by us, the total incidence of syncope is similar between genders [33].

Despite the existence of considerable knowledge regarding the occurrence of syncope in soldiers [2, 5, 13], associations between various aspects of military duty or deployment and syncope are poorly understood, with the exception of standing in ranks [2]. Future studies should also focus on this aspect.

## Conclusion

In summary, the following criteria were suggested for the ideal head-up tilt test patient:

Main criteria:

- Three or more syncopal events in the patient’s medical history
- Typical prodromal symptoms (especially vertigo, sweating or injuries in association with syncopal events).

Minor criteria:

- No further accompanying diseases
- Body height in the upper range and a body mass index in the upper normal range

Immediate head-up tilt testing might be advisable if at least one main and one minor criterion are satisfied. Admittedly, the low sample size and wide age ranges used in this proof-of-principle assessment do not provide sufficient statistical power for creating mathematical models of syncope risk. Neither the applied statistical tests nor the sample size allow for the reliable mathematical determination of criteria to identify patients who are at greater risk of syncope. Future confirmatory studies with sufficient power are warranted to confirm the suggested criteria, to ensure that the calculations are robust, and to allow for gender stratification. Such future studies should also control for underlying medical conditions, which might be critical for the outcome.

Nevertheless, head-up tilt testing should be the first diagnostic procedure used for patients with typical medical histories rather than the last test procedure undertaken. Appropriate diagnostic infrastructure and qualifications are advisable for diagnosing armed forces medical service personnel, especially in view of the importance of syncope within military settings and with respect to aviation safety [2, 5, 13, 14].

## Availability of supporting data

All relevant data are presented in the paper.

## Abbreviations

BMI: body mass index
ECG: electrocardiogram
ECG: electrocardiographic
POTS: Postural tachycardia syndrome
SD: Standard deviation
